# Connectivity in deep brain stimulation for self-injurious behavior: multiple targets for a common network?

**DOI:** 10.3389/fnhum.2022.958247

**Published:** 2022-08-24

**Authors:** Petra Heiden, Daniel Tim Weigel, Ricardo Loução, Christina Hamisch, Enes M. Gündüz, Maximilian I. Ruge, Jens Kuhn, Veerle Visser-Vandewalle, Pablo Andrade

**Affiliations:** ^1^Department of Stereotactic and Functional Neurosurgery, Faculty of Medicine and University Hospital Cologne, University of Cologne, Cologne, Germany; ^2^Department of Neurosurgery, Faculty of Medicine and University Hospital Cologne, University of Cologne, Cologne, Germany; ^3^Department of Psychiatry and Psychotherapy, Faculty of Medicine and University Hospital Cologne, Cologne, Germany; ^4^Department of Psychiatry, Psychotherapy and Psychosomatic, Johanniter Hospital Oberhausen, Oberhausen, Germany

**Keywords:** self-injurious behavior, aggressiveness, deep brain stimulation, connectivity, psychosurgery

## Abstract

Self-injurious behavior (SIB) is associated with diverse psychiatric conditions. Sometimes (e.g., in patients with autism spectrum disorder or acquired brain injuries), SIB is the most dominant symptom, severely restricting the psychosocial functioning and quality of life of the patients and inhibiting appropriate patient care. In severe cases, it can lead to permanent physical injuries or even death. Primary therapy consists of medical treatment and if implementable, behavioral therapy. For patients with severe SIB refractory to conventional therapy, neuromodulation can be considered as a last recourse. In scientific literature, several successful lesioning and deep brain stimulation targets have been described that can indicate a common underlying neuronal pathway. The objectives of this study were to evaluate the short- and long-term clinical outcome of patients with severe, therapy refractory SIB who underwent DBS with diverse underlying psychiatric disorders and to correlate these outcomes with the activated connectivity networks. We retrospectively analyzed 10 patients with SIB who underwent DBS surgery with diverse psychiatric conditions including autism spectrum disorder, organic personality disorder after hypoxic or traumatic brain injury or Tourette syndrome. DBS targets were chosen according to the underlying disorder, patients were either stimulated in the nucleus accumbens, amygdala, posterior hypothalamus, medial thalamus or ventrolateral thalamus. Clinical outcome was measured 6 months after surgery and at long-term follow-up after 10 or more years using the Early Rehabilitation Barthel index (ERBI) and time of restraint. Connectivity patterns were analyzed using normative connectome. Based on previous literature the orbitofrontal cortex, superior frontal gyrus, the anterior cingulate cortex, the amygdala and the hippocampus were chosen as regions of interest. This analysis showed a significant improvement in the functionality of the patients with DBS in the short- and long-term follow-up. Good clinical outcome correlated with higher connectivity to the amygdala and hippocampus. These findings may suggest a common pathway, which can be relevant when planning a surgical procedure in patients with SIB.

## Introduction

Self-injurious behavior (SIB) is defined as a behavior where the affected individual inflicts physical injury upon him/herself (Minshawi et al., 2014). SIB is associated with diverse psychiatric disorders. In most cases it is part of the clinical appearance of the disease. In rare cases SIB is the most dominant symptom, almost solely limiting the patients’ psychosociological functioning, reducing quality of life severely and inhibiting appropriate patient care. This mostly occurs in patients with advanced autism spectrum disorder, severe Tourette syndrome or in case of acquired brain injuries (Huisman et al., 2018). It is essential to differentiate SIB of these patients from non-suicidal self-injury in patients with borderline personality disorder or psychological distress (Brickman et al., 2014). Epidemiological studies have shown that about 4% to 9% of people with intellectual or developmental disabilities exhibit SIB; within the population of autism spectrum disorder this rate is 33% to 71% (Bradley et al., 2018) and within patients with Tourette syndrome 35% (Stafford and Cavanna, 2020). In severe cases, SIB can lead to serious permanent physical damages or even death and is frequently associated with social isolation and institutionalization. Further, it can entail a high psychological and financial burden for the social environment of the affected patient (Bradley et al., 2018).

Treatment should be focused on the underlying psychiatric diagnosis if a specific disorder is diagnosed (Antonacci et al., 2008). First-line treatment for SIB consists of pharmacological therapy. Mood stabilizers like valproate or carbamazepine have shown to be efficient in the treatment of aggression (Golden et al., 2006). Atypical antipsychotics are also used off-label, most frequently risperidone, aripiprazole or olanzapine. Behavioral therapies have been proven to be effective, however, severe symptoms may prohibit appropriate psychotherapeutic methods and only few have access to specialized centers (Antonacci et al., 2008). Also, several studies report a transient improvement of the symptoms after electroconvulsive therapy (Consoli et al., 2013).

Several stereotactic techniques have been introduced in the last decades with the aspiration to help patients with severe, otherwise therapy refractory SIB as a last resort. During the 1960s posteromedial hypothalamotomy and amygdalotomy emerged as treatment options for therapy refractory pathological aggressiveness (Narabayashi and Uno, 1966; Sano et al., 1966). Both procedures showed a significant improvement of the symptoms in more than half of the patients in the short- and long-term follow-up. However severe side effects were reported in studies with amygdalotomies, including Klüver-Bucy syndrome, seizures and hemiparesis (Narabayashi and Uno, 1966; Kiloh et al., 1974). Studies with hypothalamotomies only reported mild side effects (Sano et al., 1966). Further possible lesion targets described in subsequent literature are the anterior cingulate cortex (CC) and the anterior limb of the internal capsule (Jiménez et al., 2012). With the development of deep brain stimulation (DBS) it was reasonable to apply this possibly reversable method also for patients with severe pathological aggressiveness. The knowledge gained from studies using stereotactic lesioning was applied for the selection of possible target structures. The first DBS for pathological aggressiveness targeting the posteromedial hypothalamus was described by Franzini et al. (2005) in 2005. After that, Sturm et al. (2013) published the first case describing the efficacy of DBS of the amygdala for the treatment of pathological aggressiveness. Direct comparison of the reported cases is challenging due to the different clinical outcome measurements used, nonetheless most studies report satisfying results in most of the patients (Jiménez et al., 2012; Zhang et al., 2017; Torres et al., 2021; Yan et al., 2022). In summary, there are several possible target structures along the limbic system described in scientific literature for the treatment of pathological aggressiveness. It is believed that the neuronal circuits connecting the amygdala, the hippocampus and the periaqueductal gray, control reactive aggressiveness, moderated by the ventromedial frontal cortex, including the anterior CC (Rossel and Siever, 2015; Blair, 2016). Based on the functional and structural connections between the different stereotactic target regions and the observed beneficial outcome, this might indicate a common underlying neuronal network responsible for aggressive behavior.

The first objective of this study was to report the efficacy and safety of DBS in SIB patients treated at our clinic and their long-term clinical outcome. Our second objective was to analyze the connectivity patterns and the potentially identify common networks based on their clinical outcome.

## Methods

### Patient data

We retrospectively analyzed the data of 10 patients with SIB who underwent surgery for chronic DBS at the University Hospital Cologne between 2003 and 2009. All patients suffered from severe, continuous, therapy resistant SIB for a long time period, which was the dominant symptom of their disorder. All patients were evaluated by psychiatric experts from our hospital. Surgery was offered to these patients and their families or caregivers after individual evaluation by experienced neurosurgical and psychiatric specialists of the University Hospital Cologne. The performance of stereotactic surgery followed the premise of an individual attempt of healing in otherwise treatment refractory patients. The University Hospital of Cologne Medical Ethical Committee was informed and ethical approval was obtained before each intervention.

Five patients were diagnosed with therapy refractory Tourette syndrome, three patients developed SIB after hypoxic or traumatic brain injury and two patients suffered from SIB since childhood as part of their severe autism spectrum disorder. The target for DBS surgery was chosen according to the underlying psychiatric disorder, in all cases bilaterally. In three patients with Tourette syndrome the nucleus accumbens was targeted, in one patient the ventrolateral thalamus and in one further patient the posterolateral hypothalamus. In two patients with SIB after brain injury the posterolateral hypothalamus was targeted, in the third patient the medial thalamus. One patient with autism spectrum disorder received DBS in the nucleus accumbens, the second patient in the amygdala. Targets were identified using the Atlas of the Human Brain (Schaltenbrand and Wahren, 1977) and a preoperative MRI-scan. Stereotactic planning was performed using the STP 3.5 software (Howmedica Leibinger, Freiburg, Germany). Quadripolar electrodes (Medtronic 3389 or 3387, Medtronic Plc, Minneapolis, Minnesota, USA) were implanted stereotactically-guided, the accurate location of the electrodes was confirmed using intraoperative x-rays. Stimulation parameters were gradually adjusted individually during the follow-up visits.

Clinical outcome was measured using the Early Rehabilitation Barthel Index (ERBI) and percentage of time patients stay restrained, which was assessed prior to surgery, 6 months after the procedure and at the last follow-up appointment. On the ERBI each item measures the independency of the patient in daily activities. The final score ranges from −325 to +100 points, more positive values indicating higher independency (Rollnik, 2011). Patients were classified as responders with an improvement over 20 points on the ERBI (Quinn et al., 2011) 6 months after the surgery. Low-responders were defined as patients with an improvement between 20 and 100 points, patients were classified as good-responders with an improvement over 100 points.

### Volume of activated tissue, connectivity and common activated fibers

In order to analyze the connectivity patterns, we estimated the volume of activated tissue using the following method. The preoperative T1 sequence of each patient was co-registered and normalized to ICBM 152 MNI 2009b space (Fonov et al., 2009) through a combination of linear and non-linear transformations, using Advanced Normalization Tools (ANTs^1^, Avants et al., 2008; Tustison et al., 2019). The stereotactic AC/PC coordinates of the electrodes documented during surgery were transferred to MNI space and were projected using the open-source software Lead-DBS^2^ (Horn and Kühn, 2015). Using the model described by Horn et al. (2017a), we estimated the volume stimulated by the active contacts (VTA, Volume of Tissue Activated) based on the stimulation parameters documented at the follow-up appointment 6 months past the surgery, with a general heuristic electrical field threshold of 0.2 V/mm.

Based on previous studies (Rossel and Siever, 2015; Blair, 2016; Rizzi et al., 2017) the medial and lateral orbitofrontal cortex (OFC), the superior frontal gyrus, the anterior CC, the amygdala, and the hippocampus were defined as regions of interest (ROIs). Subcortical nuclei were identified using the MNI PD25 (Xiao et al., 2017) atlas and cortical regions using the Desikan-Killiany atlas (Desikan et al., 2006). The connectivity of the VTAs to the ROIs was analyzed in a structural group connectome based on the diffusion spectrum imaging of 32 healthy adult subjects (Human Connectome Project, HCP; Setsomop et al., 2013; Horn et al., 2017b). Using TrackVis imaging software (Wang et al., 2007), we quantified the number of tracts passing through the VTAs and connecting each of them to each ROI. The VTAs for the left and right hemisphere were analyzed separately and only the connectivity to the ROI to the ipsilateral hemisphere was included in the analysis.

In a further analysis, we performed a whole-brain analysis identifying fibers associated with better clinical outcome. First, the tracts in the normative connectome which were hit by any VTA of the whole patient cohort were isolated. Then, for each tract of this subset, two groups of patients were defined: patients whose VTAs hit the tract in question, and patients whose VTAs did not hit the tract. The clinical outcomes of these two groups were then compared using Mann-Whitney-U test and an approximate z-value was calculated and attributed to the tract in question. Negative z-scores are associated with clinical worsening, while positive ones to clinical improvement. This analysis was performed iteratively on all tracts of the subset using custom-built MATLAB routines (version 2020b, The Mathworks Inc., Natick, Massachusetts, USA).

### Statistical analysis

All data were analyzed using SPSS (IBM Corp. Released 2020. IBM SPSS Statistics for Macintosh, Version 28.0. Armonk, New York, USA). Each brain hemisphere was analyzed separately. In the absence of normal distribution determined using the Kolmogorov–Smirnov test, the Spearman rank’s correlation was conducted between the connectivity parameters of each VTA with the individual ROIs and the clinical outcome. To compare clinical outcome at different time points the Wilcoxon test was conducted. p-values under 0.05 were considered significant. The data supporting the findings of this study, such as the DBS MRI datasets, are not publicly available due to data privacy regulations, but are available from the corresponding author upon reasonable request.

## Results

### Clinical outcome

Demographic and clinical data of the patients prior to surgery is summarized in Table 1. The mean age at the date of the surgery was 30.9 years (SD ± 11 years), ranging from 13 to 45 years. On average SIB persisted for 17.1 years prior to surgery (SD ± 14.7 years). Seven patients were males and three females. The mean ERBI at the time of the surgery was −54.5 points (SD ± 74.1). In all patients, there was a significant improvement 6 months after the surgery, with a mean ERBI of 38.5 points (SD ± 72.62; *z* = −3.75, *p* < 0.001). One patient was classified as a non-responder, four patients as low-responders and five as good-responders. There was also a significant reduction of the mean duration of restraint from 65% prior to surgery (SD ± 46.2%) to 11.5% (SD ± 15.9%) of the day 6 months after the intervention (*z* = −3.09, *p* < 0.001). The individual clinical course of each patient is displayed in Table 2.

**Table 1:** Demographic table of the patients including stimulation parameters associated with the best clinical results 6 months past the surgery.

	**Gender**	**Age at surgery (years)**	**Duration of symptoms (years)**	**Diagnosis**	**Target**	**Stimulation Parameters**
Patient 1	Male	45	40	Tourette syndrome	Posterior hypothalamus	3.5 V, 120 μs, 130 Hz C+, 1-, 2-
Patient 2	Male	24	6	Tourette syndrome	Nucleus accumbens	6.5 V, 120 μs, 130 Hz C+, 0-, 1-, 2-, 3-
Patient 3	Female	40	1	Hypoxic brain injury	Nucleus fasciculosus thalami	6.5 V, 90 μs, 130 Hz C+, 0-, 1-, 2-, 3-
Patient 4	Female	40	24	Tourette syndrome	Nucleus accumbens	4.5 V, 180 μs, 130 HZ C+, 0-, 1-
Patient 5	Male	47	32	Tourette syndrome	Nucleus accumbens	5.0 V, 150 μs, 60 Hz 1 +, 2-
Patient 6	Male	46	40	Autism spectrum disorder	Nucleus accumbens	6.0 V, 90 μs, 145 Hz C+, 1-, 2-, 3-
Patient 7	Male	25	22	Tourette syndrome	Ventrolateral thalamus	3.0 V, 120 μs, 130 Hz C+, 1-, 2-
Patient 8	Female	25	7	Traumatic brain injury	Posterior hypothalamus	2.8 V, 120 μs, 130 Hz C+, 0-, 1-
Patient 9	Male	24	0.5	Hypoxic brain injury	Posterior hypothalamus	5.5 V, 180 μs, 130 Hz C+, 1-, 2-, 3-
Patient 10	Male	13	10	Autism spectrum disorder	Amygdala	6.5 V, 90 μs, 130 Hz C+, 0-, 1-, 2-, 3-

Stimulation parameters were symmetrical in all patients.

**Table 2:** Summary of the clinical outcome and complications of each patient.

	**Initial ERBI**	**Improvement in ERBI after 6 months**	**Comments**
Patient 1	−30 points	0 points	No effect on SIB, discontinued the therapy after 7 years
Patient 2	−120 points	220 points	
Patient 3	−120 points	50 points	Discontinued follow up appointments
Patient 4	−65 points	165 points	Removal of the system after 1 month because of infection, reimplantation after 3 months. Discontinued the therapy after several years due to deterioration of health.
Patient 5	45 points	55 points	Multiple modifications in the stimulation parameters initially, including high frequency stimulation. Removal of the system after 5 years because of chronic infection. Reimplantation after 6 years for thalamic stimulation.
Patient 6	50 points	50 points	
Patient 7	45 points	55 points	
Patient 8	−105 points	120 points	Removal of the system after 3 months because of infection, reimplantation after 3 years
Patient 9	−135 points	125 points	
Patient 10	−110 points	160 points	Discontinued follow up appointments

Long-term follow-up after more than 10 years could be conducted in six patients. From the four patient who did not complete the long-term follow-up, two patients initially participated in follow-up appointments, however eventually did not visit our clinic anymore and could not be contacted *via* telephone or email. Two patients were excluded due to termination of the stimulation after several years because they did not benefit from the stimulation anymore. On average the last follow-up appointment was 14.7 years after the initial surgery (SD ± 2.3 years). The mean ERBI at the long-term follow-up was slightly lower than 6 months after the intervention, however the difference was not statistically significant (mean ERBI at long-term follow-up: 36.7, SD ± 74, *z* = −1.86, *p* = 0.063). Nonetheless, there was still a significant improvement when compared to the preoperative ERBI (*z* = −2.87, *p* = 0.002). The average time of restraint also increased in the long-term follow-up to 16.67% (SD ± 24.61%) of the day, however, there was still a significant difference to the pre-operative parameters (*z* = −2.59, *p* = 0.01).

### Complications

Three patients had an infection of the DBS system, in one patient it occurred after 1 month, in the second patient after 3 months and in the third patient 5 years after the initial surgery. In all three cases a complete removal of the DBS system was performed. All three patients underwent additional surgery to reimplant the system at a later stage. Leads were placed at the same location in patients where the electrodes were removed after 1 and 3 months. For the third patient the target was changed from nucleus accumbens to the thalamus (centromedian nucleus/ventroralis internus). No further complications or permanent side effects were reported.

In both patients who underwent revision surgery within the first year, 6 months follow up data was collected 6 months after the reimplantation of the system.

### Neuroimaging analysis

**Figure 1:**
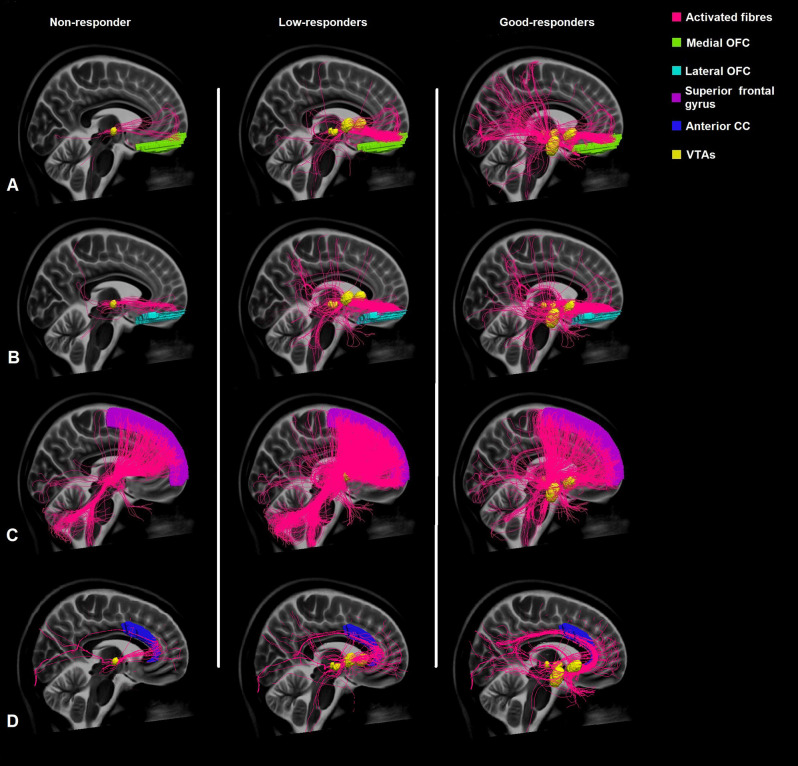
Comparison of the connectivity of the VTAs of non-responders, low-responders, and good-responders to the medial orbitofrontal cortex (medial OFC) **(A)**, lateral orbitofrontal cortex (lateral OFC) **(B)**, superior frontal gyrus **(C)**, and the anterior cingulate cortex (anterior CC) **(D)** based on normative connectome.

The proportion of tracts connecting the VTA to each ROI in all tracts activated by the VTA was calculated in the HCP normative connectome. There was a moderate correlation between the mean percentage of tracts connecting the VTAs to the medial OFC and the clinical improvement measured with the ERBI 6 months after the surgery (mean 5.21%, SD ± 8.72%, *r_s_* = 0.37, *p* = 0.109; Figure 1A), however it was not statistically significant.

There was also no significant correlation between the proportion of tracts connecting to the lateral OFC (mean 3.89%, SD ± 4.91%, *r_s_* = 0.26, *p* = 0.26; Figure 1B) or the superior frontal gyrus (mean 18.87%, SD ± 15.97, *r_s_* = −0.416, *p* = 0.068; Figure 1C) and the clinical outcome.

The portion of tracts connecting the VTAs to the anterior CC moderately correlated with the clinical outcome, however this correlation was not statistically significant (mean 2.41%, SD ± 3.93%, *r_s_* = 0.383, *p* = 0.96; Figure 1D).

**Figure 2:**
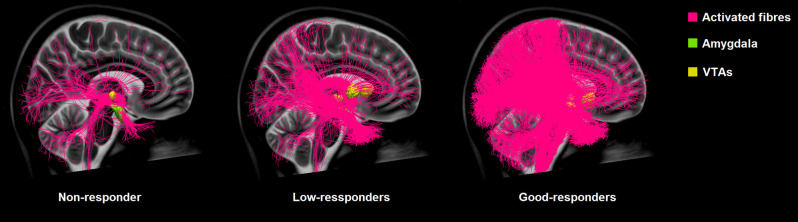
Comparison of the connectivity of the VTAs of non-responders, low-responders, and good-responders to the amygdala based on normative connectome.

Our data could show a significant moderate correlation between the clinical improvement measured on the ERBI and the percentage of tracts connecting to the amygdala (mean 12.04%, SD ± 14.19, *r_s_* = 0.478, *p* = 0.033; Figure 2).

**Figure 3:**
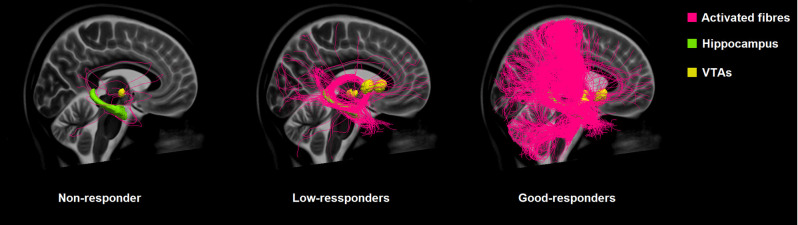
Comparison of the connectivity of the VTAs of non-responders, low-responders, and good-responders to the hippocampus based on normative connectome.

There was a stronger, also significant correlation between the clinical outcome and the proportion of tracts connecting to the hippocampus (mean 2.35%, SD ± 4.75, *r_s_* = 0.574, *p* = 0.008; Figure 3).

**Figure 4:**
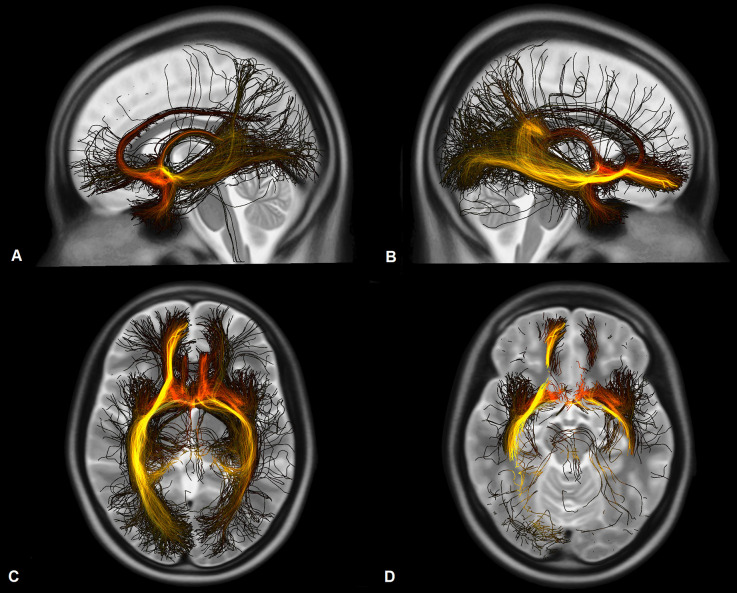
Fibers activated by the VTAs and associated with a better functional outcome of the patients measured on the ERBI in sagittal view from the left **(A)**, from the right **(B)**, in axial view on the level of the thalami **(C)**, and on the level of the hippocampus and the amygdala **(D)**. Darker colors represent a stronger correlation to the clinical outcome.

**Figure 5:**
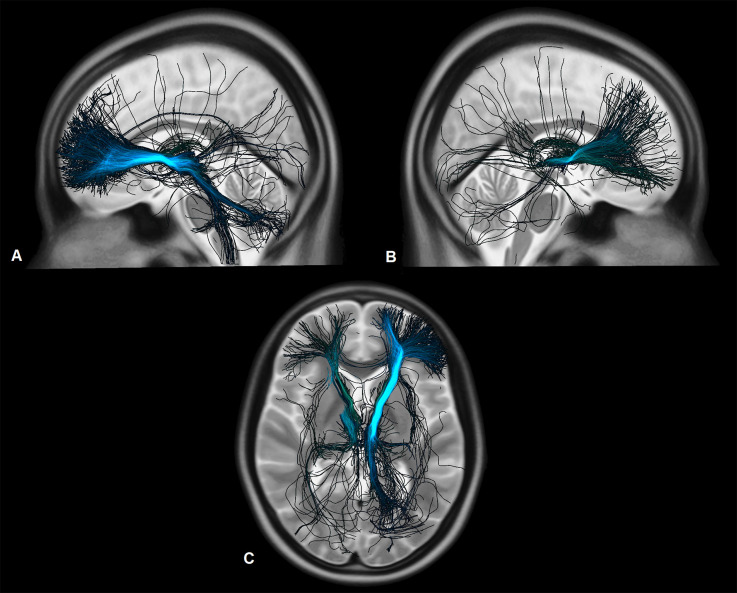
Fibers activated by the VTAs and associated with a worse functional outcome of the patients measured on the ERBI in sagittal view from the left **(A)**, from the right **(B)**, and in axial view on the level of the thalami **(C)**. Darker colors represent a stronger correlation to the clinical outcome.

Connectivity patterns of the VTAs to the whole brain weighted by clinical improvement of the patients are displayed in Figures 4 and 5. Tracts associated with better clinical improvement connected the VTAs to the amygdala, hippocampus and to the CC on both sides. Tracts associated with worse clinical outcome showed stronger connectivity to the superior frontal gyrus. In the OFC, activated fibers connecting to the medial part were associated with a greater clinical improvement, while fibers connecting to the lateral OFC were associated with less improvement measured on the ERBI.

## Discussion

In this retrospective study we analyzed the clinical course of 10 patients with diverse psychiatric disorders under DBS, which all suffered from severe, chronic, therapy refractory SIB. Primary outcome was the improvement of functionality measured on the ERBI. As a further representative measurement for the psychosocial functionality of the patients we compared their time of restraint.

Our study showed a significant improvement in the functionality and time of restraint in SIB patients treated with DBS after 6 months as well as in a long-term follow-up with over 10 years of stimulation. In this retrospective analysis, 9 out of 10 patients reached satisfactory results with DBS, whereas one patient was a clear non-responder. A further patient was explanted due to loss of effect of the stimulation after several years of profiting from DBS. These results fall in line with the clinical outcome reported in the literature (Torres et al., 2021; Yan et al., 2022), however direct comparison is not possible due to different outcome measures. In our patient group, there was no further improvement in the long-term follow-up, rather a slight deterioration compared to the follow-up 6 months after the intervention. There was still a significant improvement of the clinical status in the long-term follow-up in comparison to the baseline. Three out of 10 cases needed additional surgery because of an infection of the DBS-system were reported, which resulted in additional surgeries for the patients. All patients did well after re-implantation and suffered no permanent complications. This infection-rate is higher than in other DBS-studies, reporting a risk of infection of 5% (Kantzanou et al., 2021), probably due to the complex care and specific clinical status on account of skin wounds of patients suffering from SIB.

Analysis of the connectivity patterns using normative connectome showed a significant positive correlation between clinical improvement and the strength of connectivity to the hippocampus and the amygdala. Further, fibers associated with better clinical outcome were shown to be connected to the amygdala and hippocampus and to the CC as well. Activated fibers projecting to the prefrontal cortex overlap the medial forebrain bundle as described by Coenen et al. (2018), these fibers are mostly associated with reward-associated behavior and motivation. Interestingly fibers associated with less improvement in the ERBI after 6 months organize in a more dorsal pathway which also overlaps the common pathway for deep-brain-stimulation in obsessive-compulsive disorder, as described by Li et al. (2020). Fibers which were associated with a better clinical outcome organize in a more ventral circuit and project more to the medial OFC.

Our results suggest a common underlying neuronal network which is stimulated at different areas in these patients, resulting in reduction of SIB. Whole-brain analysis also indicates a clear differentiation of this network from the common pathway previously identified for the treatment of obsessive-compulsive disorder. Identification of a common pathway would be especially helpful in these patients as they suffer from different underlying disorders, which should be also considered during the planning of a DBS procedure. For example, in patients with Tourette syndrome and SIB it might be beneficial to consider underlying pathways both in regard to the underlying neuropsychiatric condition and the auto-mutilative behavior. Previous studies showed that stronger connectivity to the supplementary motor area (SMA) and preSMA correlate with better tic-reduction (Andrade et al., 2020), thus a target affecting the motoric pathways and also with strong connectivity to the amygdala and hippocampus might be most beneficial for Tourette patients suffering from SIB.

Activated fibers associated with a worse clinical outcome projected to the superior frontal gyrus and the lateral OFC. However, it is important to note, that these fibers were not correlating with an increase of SIB, as none of the patients had a worse clinical outcome in compare to the baseline parameters at the follow-ups. Thus, these fibers probably have no association with the modulation of SIB.

An important limitation of this study is, that it analyses a symptom in a diversity of underlying psychiatric conditions, which makes the comparison and unification of these patients difficult.

Regarding the method of neuroimage analysis, it is important to remark, that traumatic and hypoxic brain injuries are associated with altered structural and functional connectivity (Hayes et al., 2016; Smyser et al., 2019), therefore using a normative connectome when analyzing these patients might be misleading. Structural abnormalities have been also reported in the socio-emotional circuits of patients with autism spectrum disorder (Ameis et al., 2011) and studies also show an altered functional and structural connectivity in Tourette syndrome (Worbe et al., 2012; Cheng et al., 2014; Heiden et al., 2021). Analysis based on patient specific diffusion imaging data would be more suitable considering this patient collective. A further limitation is the low number of subjects in this study, which has a considerable effect on the interpretation of the clinical and neuroimaging results. A multi-center study involving larger patient groups would be necessary to further isolate neuronal networks which contribute to the reduction of SIB. However, the diversity of underlying psychiatric conditions and comorbidities of patients with SIB might complicate such an analysis. Randomized-controlled studies, although potentially of a high scientific value, are less likely to be successful in this particular patient group because of the complicated care, ethical issues related to informed consent, and lacking compliance of this patient population.

## Conclusion

In this study, we reported an improvement of the psychosocial functionality in patients with diverse psychiatric conditions with severe SIB using DBS in diverse anatomical targets. We showed a significant correlation between the clinical improvement and connectivity patterns with higher number of activated tracts to the amygdala and the hippocampus bilaterally. These findings suggest the presence of a common underlying neuronal network for SIB between these targets, which can eventually assist when considering a surgical procedure for treatment refractory patients with diverse underlying conditions and comorbidities.

## Data Availability Statement

The raw data supporting the conclusions of this article will be made available by the authors, without undue reservation.

## Ethics Statement

Ethical review and approval was not required for the study on human participants in accordance with the local legislation and institutional requirements. Written informed consent for participation was not required for this study in accordance with the national legislation and the institutional requirements.

## Author Contributions

PH, VV-V, and PA: conceptualization. PH, DW, RL, CH, EG, MR, JK, and PA: investigation. PH, DW, RL, and PA: methods. PH and PA: writing—original draft. PH, DW, RL, CH, EG, MR, JK, VV-V, and PA: writing—review and editing. All authors contributed to the article and approved the submitted version.

## Funding

We acknowledge support for the Article Processing Charge from the DFG (German Research Foundation, 491454339).

## Footnotes

^1^ http://stnava.github.io/ANTs/

^2^ https://www.lead-dbs.org/
